# Supplementary material for: Are carbon emissions trading and green financial instruments synergistic? -Comprehensive quantitative research based on content analysis
Source: PLoS One. 2024 Mar 7;19(3):e0298601. doi: 10.1371/journal.pone.0298601 (PMC10919601; doi:10.1371/journal.pone.0298601)
Supplement: S1 Data — (ZIP) [file pone.0298601.s001.zip › Data/地级市Z_F工作报告.zip!/╔╜╬≈/┬└┴║╩╨/└┤╫╘╣1⁄2╓┌║┼í╛┬φ┐╦╩2╛▌═°í┐_2012.docx]

各位代表：

现在，我代表市人民政府向大会作工作报告，请予审议，并请市政协委员和其他列席人员提出意见。

2011年工作回顾

2011年是“十二五”开局之年。一年来，在市委的坚强领导下，我们深入贯彻落实科学发展观，着力推动转型跨越发展，经济社会发展和各项工作都取得了新的成绩。

一、主要指标超额完成。地区生产总值完成1131亿元，增长14%；财政总收入完成276.93亿元，增长41.15%，一般预算收入完成100.51亿元，增长37.76%；固定资产投资完成543.7亿元，增长37.5%；社会消费品零售总额完成261.1亿元，增长17.3%；城镇居民人均可支配收入完成17428元，增长14.1%；农民人均纯收入完成4743元，增长21.9%；居民消费价格总水平上涨4.6%；城镇登记失业率控制到1.9%。万元生产总值综合能耗、工业增加值用水量和主要污染物排放量等控制在计划之内。

二、农村经济稳定增长。强农惠农富农政策落实到位。“三农”投入达到86.76亿元，增长41.94％。粮食生产连续七年丰收，达到10.6亿公斤。植树造林76.06万亩；栽植以核桃为主的干果经济林59.3万亩，干果销售收入突破16亿元。农田水利建设进一步加强，新增灌溉面积4.2万亩。农产品加工业继续发展，加工量达到110万吨。龙头企业销售收入达到40.2亿元。完成了221个新农村重点村、11个集中连片示范区建设和62个贫困村整村推进任务。新转移农村劳动力5.26万人。

三、工业转型步伐加快。规模以上工业增加值完成811.4亿元，增长18.4%，工业对经济增长的贡献率达到81.8%。煤炭资源整合后续工作深入推进，吕梁整体进入“大矿时代”。焦化、钢铁等行业的整合重组全面启动，太钢岚县项目建设加快，杏花村酒业集中发展区建设如期推进。环境质量明显改善，节能减排任务如期完成。全市二级以上天数达到4575天（包括离石区在内的13个县城的总天数），同比增加134天，空气质量优良率达到97.8%。

四、“三项整治”成效显著。大力开展以环境卫生、街道装饰、交通秩序为重点的“三项整治”活动，城乡面貌发生深刻变化。全市城镇化率提高1.5个百分点。13个县(市、区)成功创建省级卫生城市。

五、基础建设取得突破。吕梁民用机场、中南出海通道、吕临支线、太兴铁路紧张建设,环城高速、临离高速、岢临高速、黄河大桥等高速路桥项目进展顺利，县、乡公路改造取得突破，农村街巷硬化完成14854公里。柏叶口水库主体工程全部完工，离石千年水库大坝基本完成填筑任务。一批电源电网工程相继建成，发展制约有效缓解。

六、社会事业全面进步。教育事业持续发展，义务教育全面提升，高中阶段毛入学率达到92.36%，公办职业高中实现免学费入学，高等教育质量稳步提高。办学条件进一步改善，建成城区幼儿园23所，一批新建高中投入使用。医药卫生体制改革成效显著，基本药物制度在基层医疗机构实现全覆盖，新型农村合作医疗参合率达到97.25%，县级人民医院、中医院技术水平和服务能力进一步提升。文化事业繁荣发展，全市文化馆、艺术馆、图书馆、美术馆全部免费向公众开放，12个省级非遗项目申报成功，一批自创文艺作品获得全国大奖。强化科技、人才支撑，组织实施科技攻关项目33个，消化吸收科技成果近20个，争取国家、省级星火、农业攻关项目14个。稳定和扩大就业，新增城镇就业5万人，下岗失业人员再就业1.59万人。

七、安全形势持续好转。严格落实企业安全生产和政府安全监管两个主体责任，深入开展以煤矿为重点的各行业各领域的安全生产专项整治行动，一大批隐患得到治理，各类安全生产事故起数和死亡人数均较上年大幅减少，没有发生一次重特大事故。

八、民主法制和精神文明建设进一步加强。完善重大决策规则和程序。自觉接受人大和政协的监督，积极支持各民主党派、工商联、无党派人士参政议政。共办理人大代表建议167件、政协委员提案555件。扎实推进“法治吕梁”建设，“六五”普法全面启动，法制建设取得进步。不断完善基层民主建设，第九届村委会换届顺利完成。廉政建设成效明显，审计和监察工作进一步加强。深化群众性精神文明创建活动，公民思想道德素质和社会文明程度有了新的提高。

在这里，我向大会郑重报告，2011年无论是主要指标，还是主要工作，都圆满完成了预定目标任务，实现了“十二五”良好开局！

各位代表，取得这样的成绩,是省委、省政府坚强领导、亲切关怀的结果，是市委统揽全局、科学决策的结果，是市人大、市政协支持配合、有效监督的结果，是全市广大干部群众同心同德、团结奋斗的结果，是社会各界鼎力支持、无私奉献的结果。在此，我代表市人民政府，向全市人民，向驻吕解放军指战员、武警官兵和公安民警，向各民主党派、人民团体和所有关心支持吕梁发展的朋友，向尽心履职的各位人大代表和政协委员，表示崇高的敬意和衷心的感谢！

在充分肯定成绩的同时，我们应当清楚地看到存在的问题。一是产业结构不合理。主要是一产太弱，三产太小，二产独大。二是经济发展不平衡。我们既有全省财政收入最高的县，又有全省倒数的县，财政收入最高的县是最低的县的63.7倍。可用财力较低，一般预算收入仅占到财政总收入的36%，在全省各市占比最低。三是贫富收入差距大。城乡居民收入与财政收入增速差距呈现逐年拉大趋势，多数人的低收入与少数人的高收入形成很大的悬殊。四是农民收入增加难。农民收入的增长基础并不牢靠，增收门路并不宽广，农民增收成为难度最大的事情。五是生态环境很脆弱。地表破坏和挖煤漏水使我市本已脆弱的生态环境更加窘迫。此外，政府工作还存在一些缺点和不足，管理和服务水平有待提高，廉政建设仍需加强。这些都是发展中遇到的问题，我们将采取更加有力的措施，下更大的力气切实加以解决。

2012年工作安排

2012年是实施“十二五”规划承前启后的关键之年，也是全面贯彻落实省第十次党代会和市第三次党代会精神的重要一年。做好今年工作，意义十分重大。

今年政府工作要全面贯彻落实省委十届二次全会以及市委三届二次全会精神，坚持科学发展主题、加快转变经济发展方式主线和稳中求进工作总基调，认真做好扶贫攻坚、资源转化两篇文章，正确处理好煤与非煤、经济社会发展与民生改善、转变经济发展方式与增强内生活力三大关系，把握打基础、利长远、惠民生的总体要求，在项目推进、城市建设、安全稳定等十个方面实现新突破，保持全市经济平稳较快发展和社会和谐稳定，以优异成绩迎接党的十八大胜利召开。

经济社会发展主要预期目标是：地区生产总值1400亿元，增长15％；财政总收入340亿元，增长23％；一般预算收入125亿元，增长25％；固定资产投资700亿元，增长30％；社会消费品零售总额305亿元，增长17％；进出口总额10亿美元，增长11％；城镇居民人均可支配收入20000元，增长12%；农民人均纯收入5454元，增长15％；城镇新增就业岗位3.5万个，城镇登记失业率控制在4.22%以内；居民消费价格总水平涨幅控制在4%左右。这些指标是指导性的，可以根据形势变化适当调整。

约束性指标是：万元生产总值综合能耗下降3.5%，万元生产总值二氧化碳排放量下降3.7%，二氧化硫、化学需氧量、氨氮排放量分别下降2％、1.3％、1％，氮氧化物排放量与上年持平，烟尘、粉尘排放量均下降3％，万元工业增加值用水量下降3.5％。这些指标是硬任务、硬指标，必须完成。

全面做好今年的工作，必须坚持“打基础、利长远、惠民生”这一要求，统筹兼顾，协调推进。

我们要筑牢打基础的思想。“求木之长，必固其本；欲流之远，必浚其源。”高楼万丈平地起。打基础，就是要立足吕梁市情，正视发展差距，加强薄弱环节，不浮躁，不骄傲，沉下去，扑下身，做实在的事，干有效的工，真正打牢协调发展、均衡发展、科学发展、安全发展的基础。

我们要坚持利长远的方略。“不谋万世者，不足谋一时”。利长远，就是不能急功近利，不能只顾眼前，而是要干长远、谋长远、利长远，也就是要加快经济发展方式转变，切实提高经济发展质量，大力改善经济发展条件，努力实现更长时期、更高水平、更好质量、更可持续的发展。只有这样，我们的事业才能恒久绵长。

我们要抓住惠民生这个根本。“为政之道，以厚民生为本”。惠民生，就是要坚持把保障和改善民生作为政府工作的根本出发点和落脚点，真正做到常怀济民之心，坚守为民之责，多办利民之事，搞好“基本民生”，保障“底线民生”，关注“热点民生”，让人民群众更多地享受改革发展的成果。

具体讲，今年要做好十方面的工作：

一、扶贫攻坚

农业本身是弱质产业，总量小、基础薄、结构不优、产业化水平不高、农民收入低，成为我市经济社会发展的短板。特别是国家扶贫标准提高后，我市低收入人口由70多万增加到100多万，占全省的1/4，如此大面积的贫困问题解决不好，全市建设全面小康的目标就会落空。我们要抓住用好国家新一轮扶贫开发和我市继续作为国家重点扶持地区的机遇（临县、兴县、岚县、石楼、方山、中阳等6县被列为全国扶贫开发重点县，其中临县、兴县、岚县、石楼等4县被列入吕梁山集中连片特殊困难地区范围县，同时，省里的扶贫政策要覆盖所有县市区的低收入人口），采取有力措施，确保见到实效。

1、有效增加农民收入。要把农民收入翻番作为核心指标，把“一村一品”、“一县一业”作为主攻方向，深度开发特色优势产业，扩大生产规模，形成主打商品，提高农业生产的比较效益。不断提高6个省级“一县一业”基地县开发水平，在巩固扩大去年“一村一品”专业村建设成果的基础上，今年再实施200个以上“专业村”建设。实践证明，农民稳定增收的关键还是要在“种”、“养”、“加”三个方面找门路，这是最现实、最可行的选择和办法。在种植上，一是要大力发展设施蔬菜，今年全市要下决心新发展设施蔬菜2万亩，不仅能使农民增加收入，而且还能使居民吃到新鲜蔬菜。二是继续发展核桃经济林，年内新植核桃50万亩，使全市核桃达到300万亩。三是积极发展苗木经济，扶持绿化苗木、景观苗木和干果苗木尽快形成产业规模，努力把文水、汾阳、交城、岚县、方山、石楼等县（市）建成全省乃至西北地区重要的优质苗木生产基地。在养殖上，重点抓好规模化养鸡、养牛、养羊、养猪项目，四大养殖每县至少要有一种形成规模，养殖户达到农户总数的五分之一以上。大力支持新希望集团、大象集团在我市兴办更多的养殖基地。今年大象集团要分别在离石、柳林、临县上马建设3个养肉鸡4000万只、12个种鸡存栏10万套的大型养殖场，使全市养鸡数量在近期达到“保二争三”，即保证2亿只、争取3亿只，带动1.5万户农民增加收入。交城万通养牛今年也能超过1万头，可使2000多农户从中受益。在养猪、养羊上也要有大的突破，建成汾阳荣璋万头绿色有机猪场等一批重点养殖企业。全市今年要新建100个标准化示范养殖小区。在加工上，扶持红枣烘干加工，在沿黄四县建设一批集中加工园区，使红枣烘干率达到60%以上。在孝义开工建设投资5亿多元的核桃加工项目。做大做强畜禽加工企业，大象今年要分别建设3个屠宰4000万只、饲料24万吨的加工企业，并尽快建成孝义大象1亿只肉鸡加工和50万吨饲料项目。孝义铭信也要达到4000万只肉鸭养殖加工规模。全市今年要新上投资亿元以上的农业产业化项目5个，新建投资千万元以上的大型加工企业20个，使全市农产品年加工总量达到120万吨，龙头企业年销售收入达到45亿元以上，以这些大项目来带动千家万户的致富。同时抓好销售工作，培养经纪人队伍，发展农村专业合作化组织，办好汾阳核桃会展中心，开好国际核桃大会。在劳务上，提高劳务输出组织化程度，加大转移培训力度，打响“吕梁劳务”品牌，促进农民工资性收入稳步提高。今年培训15万人，转移5万人，使转移总量达到70万人，劳务收入占到农民人均纯收入的60%左右。

2、切实改善农村条件。一是努力增加移民数量。进一步加大易地扶贫搬迁力度，注重解决好新村占地、生产门路、生活保障三个关键问题，下决心把实际居住人口不足150人、不具备基本生存条件的山庄窝铺人口迁出去，今年扶贫移民要达到2万人以上。只有移民，才能彻底拔掉穷根。二是继续推进生态建设。抓好30万亩柠条绿化重点工程，继续实施20万亩天然林保护、通道绿化、荒山绿化和城郊绿化等其他造林绿化工程，加上50万亩核桃经济林，今年共计完成植树造林100万亩，使我市森林覆盖率提高一个百分点以上。三是积极发展水利灌溉。今年要启动实施“山区千井灌溉富民工程”，即在贫困山区每年新打机井200眼，力争“十二五”期间达到1000眼，增加灌溉面积24万亩。紧紧抓住全省“大水网”中部引黄工程建设机遇，从根本上解决工程涉及我市9县的缺水问题。加快建设柏叶口水库、千年水库等水源工程和龙门渠引水到交城的工程，力争碛口水利枢纽工程年内有重大进展。加快西山沿黄灌溉工程建设，其中柳林上庄、兴县张任碧、石楼曹家垣、临县马家湾、高家湾等5处续建工程及新开工的临县郭家洼灌溉工程年内通水受益。开展以汾河、文峪河、三川河及其他跨县域河流为主的河道治理，提高防洪能力，提升景观效果。同时完成中低产田改造26万亩，秸秆还田100万亩，测土配方施肥400万亩，努力实现“十二五”末人均1亩水浇地、1亩高产稳产旱作农田的目标。四是扎实搞好新农村建设。在巩固已经实施的1244个推进村的基础上，今年再完成重点推进村350个，同时建设高标准新农村100个和连片示范区11个。要高度重视现代科技对农业生产的支撑引领作用，增加农业科技投入，健全基层农技推广体系，加快农业机械化步伐，逐步实现农业机械化、科技化。

3、不断强化农业保障。坚持改革的理念、“多予”的原则，首先，加大财政对“三农”的投入力度，今年市本级财政在十分困难的情况下，预算新增“三农”支出2亿多元，用于农业项目的贴息或以奖代补，重点支持农业的发展。其次，大胆探索增加农民收入、规避农业风险、激发农村活力的新办法、新机制。从今年开始，按照综改试验区先行先试的要求，逐步探索尝试两方面试点：一是全面启动种植业保险，由市、县政府给予补贴，以正常年景产值为基准，将种植业自然灾害损失纳入保险赔付范围，使广大农民从经济价值的角度实现旱涝保收，真正摆脱靠天吃饭的被动局面。逐步启动养殖业保险，着力降低养殖户风险。二是建立以煤补农直补基金，试行从吨煤中收取一定资金建立专项基金，由政府直接补贴给因煤矿开采造成塌陷、漏水等致贫的农民，使农民的收入得到一定保障，从根本上缓解村矿矛盾和贫富悬殊问题，同时也把煤矿从纠纷中解脱出来。在扶贫攻坚上，我们还要坚持多轮驱动，做到政府主导、分级负责、多方投入、合力推进，充分发动全社会参与扶贫，组织行业扶贫和企业扶贫，做好机关定点扶贫，搞好干部下乡住村活动，形成扶贫攻坚的强大合力。通过以上努力，力争达到国家要求的“两不愁三保障”（不愁吃、不愁穿，义务教育、基本医疗、住房有保障）；力争达到省要求的到“十二五”末农民人均纯收入上万元，基本消除人均5000元以下的县，现有贫困人口一半以上实现脱贫以及全市农村都达到重点村建设标准、基本完成山庄窝铺移民搬迁的目标。

各位代表，“三农”工作事关全局，扶贫攻坚任重道远。老百姓是天，老百姓是地，让老百姓富裕是各级政府的职责所在，也是我们永恒的追求和工作目标。老百姓一天不能稳定脱贫，我们就一天不能懈怠，一天不能松劲。扶贫攻坚是惜民力、集民智、解民困的重大工程，是顺民意、谋民利、得民心的重大任务，我们一定要含着感情抓，带着热情干，怀着激情办，真正找对路子，寻好法子，扑下身子，坚决走出一条吕梁脱贫致富的康庄大道！

二、大上项目

项目是发展的支撑和依托，上大项目大发展，上小项目小发展，不上项目不发展，已经是被实践证明了的。为此，我们必须毫不动摇地坚持项目建设战略。

1、紧紧咬住骨干项目。一是做大煤焦铝铁，固本强基础。煤炭产业是吕梁经济的支柱和优势，将长期保持有利地位，对全市经济具有持久的基础作用、拉动作用和稳定作用，必须进一步做大做强。近期全市要形成以40多对100万吨以上的矿井为主体、以4对1000-1500万吨的矿井为“旗舰”的大矿格局。特别是今年要建成兴县华电1000万吨、临县霍州煤电1000万吨矿井，兴县西山第二个1500万吨矿井要达产，同时加快总产能近1000万吨的交口5个露天矿建设，使全市年内煤炭总产量达到1.5亿吨以上，销售收入突破千亿元，实现税费250亿元。焦炭业要利用当前全省焦炭整合和市场低谷的机遇，抓紧产能置换，兴办集中园区，开工建设500万吨焦炉2户，整合提升 200—300万吨级焦化企业7户，巩固完善100万吨级焦化企业8户，加快孝义1500万吨新型煤化工园区建设，使全市焦炭生产能力达到4000万吨左右。铝业要加快推进兴县百万吨氧化铝厂的建设，年内完成一半以上投资；争取交口信发二期120万吨氧化铝尽快投产，企业总产能达到240万吨；孝义兴安公司二期120万吨多品种氢氧化铝年内要达产达效，企业总产能达到240万吨。这样，使全市铝产量达到近600万吨，占到全省的一半。钢铁业要继续做大中钢，加快建设1780立方米高炉，使年产量达到 350—400万吨。迅速扩张文水海威钢铁，建成1380立方米高炉，年产量达到200万吨。大力推进太钢岚县项目建设，确保年产铁精矿粉750万吨、球团200万吨，使全市钢铁年产量达到700万吨。积极争取太钢岚县500万吨炼钢项目建设。稳住了煤焦铁铝，就稳住了我市经济的基本面，就打下了扎实的基础，也就解决了我们一要吃饭、二要发展的问题，我们也就可以底气十足，腾出手来，甩开膀子，拿出钱来干许许多多的事情。二是实施“百项重点工程”，牵住“牛鼻子”。今年市政府将全力抓住10亿元以上的产业项目50多个，1亿元以上的农业项目10个，1亿元以上的服务业项目10个，重大基础设施项目20个，市本级城建项目10个，共计100个大项目，总投资5000多亿元。这100个项目是重中之重，是今年全市项目建设的王牌，其中产业项目超过100亿元投资的就有6个，如信发集团孝义铝系综合循环利用项目、森泽柳林煤矸石提取氧化铝项目，还有城建上的吕梁新城项目，基础设施上的公路建设项目等，这些项目，体量巨大，技术先进，产业完备，市场广阔。抓住了这些大项目，就牵住了吕梁大发展的“牛鼻子”。同时，可以以大带小、以大促小、大小互进，形成一个大项目促进大发展、多项目促进快发展的良好局面。

2、努力突破转型项目。一是做好煤炭转化。坚持以煤为基、以煤兴业，多条路径延伸煤、扩展煤，更好地发挥煤炭转化对全市产业结构调整的带动作用。要抓好“煤转电”。我市目前电力装机总量仅为200多万千瓦，发展严重滞后，与我市煤炭大市的地位极不相称。煤炭要转化，首先就是要上电厂。今年，柳林宏光2×30万千瓦煤矸石发电项目上半年实现调试发电，联盛2×30、交城2×30、文水2×60、汾阳2×30万千瓦等有“路条”的电厂项目年内必须开工建设。积极争取柳电三期2×60、柳林华光2×60、临县晋太4×60、临县霍电2×30、临县大唐2×30、交口华瑞2×35、孝义新阳2×30、兴县2×35、中阳大唐国际2×30、中阳联盛能源2×35、岚县华润 2×100、岚县华电4×66、交城国锦热电二期2×60万千瓦等一批做了前期工作的坑口发电项目领到“路条”或获批立项，全力争取碛口水电枢纽工程立项，在较短的时期内使全市装机容量力争达到1000万千瓦以上，提高煤炭就地转化率，做到变输煤为输电。要抓好“煤转‘化’”。努力延伸煤焦化、煤气化、煤液化等循环链条，加快建设交城宏特百万吨煤焦油深加工项目，保持国内领先地位；积极推进孝义金晖、金岩90万吨焦炉煤气合成甲醇及15万吨粗苯精制、兴县锦兴180万吨煤制甲醇和60万吨煤制烯烃、汾阳五麟50万吨煤制甲醇等一批重点化工项目建设，尽快形成焦化并举、化产引领的新格局。要抓好“煤转气”。加快开发利用临县、兴县、石楼煤层气的步伐，积极推动岚县皖北煤电20万立方米煤制天然气等一批重点项目建设。二是延伸铁铝加工。改变原材料生产的落后局面，努力向产业链条高端发展。扎实抓好交城古特金20万吨注塑机汽车配件、交城兴龙铸造16万吨全自动静压造型、岚县普明工业园区30万吨精密铸件、交口4个10万吨冶炼铸造等深加工项目，促进钢铁向铸造和装备制造业转化发展。推动氧化铝加工转化，建成交口兴华科技20万吨特种氢氧化铝深加工项目，大力开发铝型材生产，实现铝产品上档升级。三是做大白酒产业。进一步加快杏花村酒业集中发展区建设，确保年内部分项目竣工投产，使汾酒年产增加10万吨，巩固提升我市清香型白酒全国第一的地位。同时，下决心整合汾阳、文水的散酒、原酒生产，统一品牌，统一销售，形成我市白酒生产又一个“龙头”。在转型上，我们必须强力推进，那就是在全市所有煤矿推行“1+1”工程，即要求一个煤矿必须延伸一条非煤产业链或者建设一个非煤项目。同时，大力支持企业技术改造和技术进步，推动工业化与信息化深度融合，真正提高我市的资源就地转化率、传统产业循环率、新兴产业占比率和科技贡献率，以此实现我市转型的突破。

3、全力扶持高新项目。一是建设吕梁高新技术产业开发区。今年要下决心在离石建设吕梁高新技术产业开发区，设立院士工作站，成立著名学校院所联合实验室，建成大规模智能电池管理系统、大功率激光熔覆再制造、光学成像和测量系统等三大高科技产业生产和技术孵化基地，改变我市高新技术产业缺乏发展平台的状况。二是建设一批高科技项目。促进孝义晋越峰纳米超级电容电池动力源项目尽快规模化生产，加快交城国利天能科技公司年产600兆瓦太阳能聚光热发电用反射镜项目开工建设；要大力培育一批节能环保、电子信息、新材料等新兴产业，使我市高新技术产业在全省占有一席之地。三是提高循环经济发展水平。建设一批自我循环、吃干榨尽的项目，推动文水金地煤焦化电建一体化建设，完善交城夏家营循环经济园区一体化工程，加快兴县资源型循环经济园区建设步伐。四是重视支持中小微企业发展。中小微企业一般能提供70%的就业岗位和解决一半城乡居民的收入，我们要高看一眼、厚爱三分，加大扶持力度，提供优质服务，帮助解决贷款难、融资难、销售难等问题，真正形成大企业引领、小企业支撑的格局。

4、重点培育三产项目。三产服务业是我市经济社会发展的薄弱环节，要制定出台相关扶持政策，统筹发展各类服务业。我们要大力支持通信、邮政等生产性服务业，壮大拓展家政、养老等生活服务业，提升优化商贸、餐饮等传统服务业，积极开发金融、旅游等现代服务业。今年要突出抓好四项工作：一是引进连锁名店。要引进红星美凯龙、居然之家等知名连锁店落户开业，争取香格里拉、苏宁、王府井等大型企业进驻经营。同时抓好孝义沃尔玛综合商务区中心超市、汾阳西部家居商贸之都、交口双池大型农副产品交易市场和物流配送中心、文水陆宇物流园区等重点项目建设。二是发展现代金融。要大力支持国有商业银行健康发展，创造条件吸引更多的股份制银行来吕梁设立分支机构。深化农村信用社改革，有序发展村镇银行、小额贷款公司、融资性担保公司等准金融组织。开发股权投资基金等新的金融产品，引导民间资本以股权、债权形式进入实体经济。积极搭建政银企合作平台，提高存贷比例，扩大信贷投放，特别要加大对中小微企业和农业的信贷支持力度。支持一批企业直接上市融资。三是开发观光旅游。重点开发一陵三馆红色游（交城吕梁英雄纪念广场，刘胡兰纪念馆、晋绥边区革命纪念馆、红军东征纪念馆）、一沟三山生态游（庞泉沟，卦山、北武当山、云顶山）、一河三水水上游（黄河，文峪河水库、柏叶口水库、横泉水库）、一园三点文化游（汾酒文化园区，碛口、玄中寺、安国寺）及石楼黄河湾、文水苍儿会等一批特色旅游景区，要修公路、连成线，能吃住、有看点，让吕梁的旅游尽快能火起来。四是壮大集散物流。以太中银铁路、吕临支线、出海大通道、太兴铁路和公路、机场为依托，同步建设一批专用线、战略装车点和大型物流园区，使物资能装得上，拉进来，运得走，使吕梁成为承东启西的重要物流中心。

5、强力推动项目落地。要强化项目领导，落实一个项目、一名领导、一套班子制度，责任到人头、到单位，同时实行动态管理，光说不动或进展缓慢的，坚决剔出重点项目盘子，取消优惠扶持的政策措施。要力攻项目审批，抓住立项、环评、土地“三大关”，组织专门力量跑审批、要路条，使项目合法化。要严格考核奖惩，完善考核内容，细化考核办法，连线督查、随机抽查、定期检查和年终考核相结合，根据项目建设绩效，实行重奖重罚，把各级干部的精力引导到项目建设上来。

各位代表,大上项目、上大项目、上好项目，是我们吕梁多年来的成功经验，我们必须一以贯之、坚持到底，这是固本强基的工程，是长远发展的战略。项目兴则吕梁兴，工业强则吕梁强。吕梁能否平稳较快发展，关键看项目；吕梁能否可持续发展，关键看转型。所以，我们要咬定青山不放松，以非常之策、非常之力、非常之为，在项目建设和产业转型上取得更大成绩。

三、基础设施

基础设施是经济社会发展的基本条件和重要支撑，要既立足当前，又着眼长远，大力度抓紧实施和建设，尽快破解经济社会发展的“瓶颈”制约。

1、完善交通体系。吕梁机场，今年全面完成建设任务，力争实现通航。铁路方面，加快中南出海大通道、吕临支线、太兴等三条铁路的建设。启动阳双铁路（孝义阳泉曲—交口双池）筹建工作，解决交口煤炭外运的问题。在经过协商于3月10日顺利开通离石直达北京夕发朝至列车的基础上，大力争取离石—太原、离石—北京动车的开通，使群众低成本、方便快捷地出行。完善扩建吕梁火车站，增加运送旅客的能力。公路方面，今年要完成岢临高速、临离高速、市区环城高速三条高速和太佳黄河大桥、机场连接线的建设并实现通车。要开工建设太佳高速岚县、临县、方山三条一级公路连接线。积极争取西纵高速离石—交口的延伸工程和石楼连接线工程，争取交口县石口—汾阳高速公路立项。尤其要争取大武—交城国防高速公路列入全省高速公路规划并立项，使吕梁城区到太原再增加一条直线高速，不仅拉近与省会的距离，而且还可以打通庞泉沟、北武当山等旅游线路。开工建设兴县—岚县一级路，解决忻黑线运煤通道拥堵问题。开工建设交口—孝义一级路，打通交口运输瓶颈。建设北城一级汽车站。启动209国道方山峪口—离石交口段改线工程，为吕梁新城腾出空间。积极争取307国道汾阳—文水—交城段改线工程，开工建设307国道柳林城区段改线工程，还要努力争取建设几条旅游公路。尽早实现我市民航开通和县县通铁路、通高速、通一级公路的历史性飞跃，真正达到人便其行、货畅其流。

2、强化电力保障。新建吕梁北（临县白文镇）500千伏变电站1座，新建岚县袁家村、文水宜安、柳林龙花垣220千伏变电站3座，年底建成投产。配合中南铁路建设，推动兴县康宁、临县清凉山、柳林贾家垣、石楼东卫等4座220千伏变电站具备开工条件。改造提升主要输电网络，全面加强市域电力保障能力。

3、加快气化进程。加快煤层气勘探开发力度，力争年内形成5亿立方米产能。今年要完成离石城区天然气置换焦炉煤气工程，加快天然气、煤层气发电供暖步伐，尽早取消燃煤锅炉。有条件的县也要开发本地气源或利用过境天然气，尽快解决居民用气和供暖问题，提高能源利用清洁化水平。

四、城镇建设

城镇化是统筹城乡发展、扩大内部需求、促进经济增长的重要途径。我市城镇化率不达40%，在全省居下游位置，比全省平均水平低10个百分点。同时，我市城镇化又滞后工业化10个百分点，发展空间很大。大家知道，城镇化率每提高一个百分点就可拉动内需约380亿元。所以，我们一定要把城镇化建设放在更加突出的位置，加大力度，超前谋划，努力实现赶超式发展。

1、建设吕梁新城。今年，我们要着手规划建设一座吕梁新城（一期工程为吕梁学院以北、方山县大武镇以南，约10平方公里；二期工程为方山县大武镇以北、方山县峪口镇以南，约10平方公里。规划总面积计20平方公里），要一次规划、分期实施。一期工程要做到一年框架成型、三年全部入住、五年全面完善。同时继续加快离石东城新区开发，以此拉大吕梁市区框架，扩大城区面积，做大城市体量，完善城市功能，提高城市的承载力和吸附力，彻底解决城区交通拥堵、空间狭小、超出负荷、生活不便等问题，真正发挥吕梁市区域中心城市的功能。同时动工兴建吕梁大医院、大剧院、文化宫、会展中心、博览馆、图书馆、艺术馆、新高中、新初中、新幼儿园等一批重点工程，建设大武火车站、新城汽车站。要继续完善城北区域性工程，特别是火车站站前广场要加快建设，投入使用。

2、县城扩容提质。各县（市、区）要以“大县城”建设为目标，大力推进城市扩容提质，开辟一个新城区，做到高起点规划、高标准建设、高水平管理，整体提升县城品位，要放开户口，集聚人口，引导生产要素和优势资源向县城集中，建设更多的5万、10万、甚至20万人口的大县城，提高县城辐射带动作用。

3、集镇重点突破。今年起每个县（市、区）都要抓住一两个大集镇，加大投入，集中改造，形成各具特色、各显优势的区域发展中心。重点规划建设柳林留誉、临县三交、文水开栅、汾阳杏花、孝义梧桐、交口双池、方山大武等一批大型集镇，特别要在推动省委、省政府关心支持的文水刘胡兰镇的建设上取得明显进展。

4、巩固扩大“三项整治”成果。建立完善城市管理长效机制，保持环境卫生，打击私搭乱建，维护交通秩序，坚决制止回头和反弹现象的发生。

5、严格规划和搞好城镇绿化。要高度重视城市规划和规划的执行，坚决制止和打击违规建设行为。统筹考虑吕梁市区和方山、中阳、柳林3县功能定位和产业布局，加快“一市四区”的规划审批进度和同城化、一体化进程，严格控制初级能源、原材料工业，逐步搬迁高污染、高耗能企业。加大环城绿化力度，提高城市周边植被覆盖水平。市区及各县县城今年都要启动建设环城林带或森林公园，增加建成区森林覆盖率。通过以上措施，使我市城镇化率尽快达到全省平均水平。

各位代表，基础设施建设和城镇化，都是吕梁走向现代化的必由之路，都是关乎吕梁长远发展的战略性工程。我们一定要着眼未来，坚定信念，用超前的谋划、顽强的毅力和勤劳的汗水，共同创造美丽宜居的新吕梁！

五、普惠民生

民生连着民心。各级政府要牢固树立群众利益无小事、民生事业大于天的理念，千方百计谋民利，竭忠尽智解民忧。

1、抓好重点民生工作。要不折不扣地完成省政府下达的新的“五个全覆盖”工程，今年是该工程实施最后一年，要把所有没有完成的项目在今年全部完成，真正达到“幸福全覆盖”。认真抓好保障性住房建设，今年全市要完成16627套的建设任务，要赶进度、保质量，公平分配。切实保障好困难群众的基本生活，力争全年物价涨幅控制在全省平均水平以内。

2、完善社会保障体系。继续扩大社会保险覆盖面，稳步提高社会保险统筹层次和待遇水平。实现城乡养老、医疗保险“全覆盖”，失业、工伤、生育保险覆盖规定的职业人群。进一步健全最低工资标准、职工工资、城乡低保标准的正常调整机制，稳步提高城镇居民收入。提高公务员津贴补贴，落实事业单位绩效工资，努力提高公职人员收入。完善社会救助体系，统筹抓好社会福利、城乡社会救助、农村五保户集中供养等工作，积极发展慈善事业。

3、办好“方便农民五件实事”。一是每村办一所幼儿园，并配备幼儿教师，要注意充分利用农村“撤点并校”留下的空壳教室，节约投资，加快进度，方便更多的农村留守儿童和学龄前儿童有去处、有人教、有所学。二是每村建一个洗澡理发室，方便群众讲究卫生。三是每村主街道安装太阳能路灯，方便群众出行。四是每村建设一座设备齐全的磨面碾米豆腐房，方便群众生活。五是每村建设一个红白喜事厅，方便群众移风易俗，文明办事宴。这些东西看似小事，但群众离不开，群众很需要，所以我们要尽力实施，市、县两级政府要尽快筹措资金，分解任务，力争到“十二五”末把这5件方便农民的实事全部完成好。

六、社会事业

社会事业是吕梁的“短腿”，要加大投入力度，提高发展水平，弥补历史欠缺，实现协调发展。

1、促进文化大繁荣大发展。实施社会主义核心价值体系建设工程、文化惠民工程、文化产业发展工程、文化精品工程和文化体制改革工程。特别要加大宣传吕梁的力度，挖掘和弘扬“吕梁精神”，树立新形象，讴歌新风貌，让更多的人了解吕梁、认识吕梁。加强县级图书馆、文化馆、体育馆、电影院等基础设施建设，抓住万村千乡文化设施建设，逐步完善基层公共文化服务体系。加强非物质文化遗产保护和传承。鼓励文艺创作，多出精品力作。大力发展文化产业，打造吕梁文化优势企业和品牌。

2、坚持优先发展教育。促进义务教育均衡发展，大力实施薄弱学校改造计划和义务教育学校标准化建设，柳林、中阳、汾阳、交口4县（市）通过省政府义务教育均衡发展验收。全面配套建设吕梁新城区高中、初中、小学、幼儿园，保证吕梁学院附中及柳林3所、汾阳2所、岚县、临县、方山、文水各1所共计10所高中年内投入使用，使高中阶段毛入学率达到93.5%。

加快吕梁教育学院和市直中专学校整合。组建高等师范学院和综合性高等学院，加快孝义吕梁职业技术学院的建设进度，尽快投入使用。支持吕梁学院、山西医科大学汾阳学院的建设和教学。积极发展继续教育、特殊教育，促进各级各类学校全面协调发展。完善教师培养培训和考核奖惩制度，加强教师队伍建设。加强教学管理，实施素质教育，提高教学质量，以优质教育资源吸引老师、留住学生。

3、进一步加强科技和人才工作。组织技术攻关，争取在煤层气利用、煤矸石清洁回收加工以及红枣防裂果防腐烂、核桃晚霜冻害防治等关键领域取得突破。加强与知名大学和重点科研机构的联系和合作，争取建立宏特煤化工等几个国家级或省级重点实验室，提高我市科技自主创新能力。培养造就大量的创新创业人才和急需紧缺人才，大胆引进一批高端人才和拔尖人才，努力营造人才辈出、人尽其才、才尽其用的创业环境。

4、加快发展卫生事业。要新建吕梁大医院，扩建市人民医院门诊楼，完善汾阳医院急诊综合楼。各县（市、区）都要重视本级医院的现代化建设。要进一步推进公立医院改革，着力解决群众看病贵、看病难的问题。巩固扩大基本医保覆盖面，城镇居民医保和新农合补助标准提高到每人每年240元，将新农合重大疾病保障由8项扩大到20项。努力做好民政救灾福利工作。继续实施贫困残疾人康复救助，扎实做好疾病防控、妇幼卫生和爱国卫生工作。继续抓好人口计生工作，实施“三晋康家”工程，切实稳定低生育水平。关心老龄和妇女儿童事业，开展全民健身运动，大力支持军分区、武警、消防、民兵预备役等军队和国防建设，做好老干部工作，做好大学生就业工作，统筹做好统计监测、防震减灾、档案史志等工作，促进各项事业蓬勃发展。

各位代表，为群众诚心诚意办实事，尽心竭力解难事，坚持不懈做好事，是人民政府的基本职责和工作取向，我们一定要勤勉工作，狠抓落实，让人民群众生活得更加富足、更有尊严！

七、安全稳定

发展是第一要务，稳定也是第一要务。要坚持安全稳定始终先于一切、大于一切、高于一切、重于一切，防范事故，化解矛盾，促进社会和谐稳定。

1、切实加强安全生产。时刻绷紧安全生产这根弦，不允许出现丝毫的麻痹和懈怠。严格落实安全生产责任制，实行安全生产挂牌制度。不断强化安全生产基层基础工作，抓好安全质量标准化建设，强化专业技术人才培养和职工安全技能培训，突出抓好劳动用工管理和企业现场管理。把安全生产作为考核各级各部门和企业的重要内容，严格实行“一票否决制”。严肃对待事故，严格责任追究。继续坚持近年来行之有效的好经验、好做法，今年要以煤矿安全为重中之重，切实加强企业主体责任、政府及部门监管责任、基建矿监管“三大管理”工作，确保“六大员”、“五人监管小组”等煤矿安全监管措施的真正到位，搞好非煤矿山、道路交通、危险化学品、易燃易爆物品、民爆物品、食品药品、特种设备、建筑施工、消防等各行业、各领域的安全隐患治理，抓好森林防火、畜禽防疫、抗旱防汛等工作，坚决杜绝重特大安全事故，保证全市安全生产形势持续好转。

2、全力维护社会稳定。深入开展“矛盾纠纷大排查、领导干部大接访、信访问题大化解”活动，畅通信访渠道，完善长效机制，充分满足群众合理诉求，坚决维护群众合法权益。加强和创新社会管理，提高科学管理水平，完善治安防控体系，扎实抓好流动人口、重点人群、信教群众以及重点区域、信息网络的服务与管理。始终保持对重大刑事犯罪的严打态势，全力维护政治稳定、社会安定。建立健全应急管理和救援体系，完善突发事件信息通报和新闻发布制度，正确引导和依法控制舆情，营造健康向上的舆论环境。

八、综改试验

转型综改试验区建设是当前最大的优惠政策，最好的发展机遇，要以先行先试的胆略，善行善试的智慧，敢行敢试的闯劲，加快走出资源型贫困地区转型跨越发展的新路子。

1、在转型综改试验区建设上要先行先试。一是抓好试点工作。我市1个省级试点县孝义、2个市级试点县中阳、岚县以及“一市两园”（交城循环经济园和吕梁高新技术产业园区）、“一县一企”试点工作争取走在全省前列，同时要把20个标杆项目建设好，特别是要把综改2012年行动方案制订好、执行好。二是创新体制机制。在项目审批上，试行“园区式审批”、捆绑式审批和链条式审批。在土地管理上，试行市域内城乡建设用地增减挂钩和矿业用地、露天采矿用地调节使用办法；试行市域新增建设用地和补充耕地指标县际调节使用办法；试行荒山、荒坡、荒沟、废弃地市级调节使用办法。在环境容量上，推行生态环境补偿和排污权交易，试行市域范围环境容量总量控制、调节平衡与市级审批办法。在金融创新上，试行筹建“吕梁转型发展股份制银行”，解决地下钱庄泛滥和民间高利贷突出的问题。三是创建试验区。在全省贫困人口最多的临县设立“扶贫攻坚试验区”，在县域经济最为弱小的石楼县设立“脱贫脱困试验区”，在财政最困难的文水县设立“财政脱困试验区”，同时在汾阳市设立省级“杏花村汾酒保护与开发经济区”。在试验区内要实行更加灵活的政策，赋予更为完整的权力，给予更大力度的扶持，使这些区域得到快速发展，加快实现综改试验目标。四是提升工业园区。解决孝义梧桐、交城夏家营、文水百金堡、兴县循环经济开发区4个省级园区和每县1个工业园区的管理机构和职级配置问题，同时切实提高园区规划、建设、管理的现代化水平，加强园区基础设施建设，在园区规范化、现代化建设上迈出实质性步子。

2、在其他各项改革上要敢行敢试。大力推进事业单位分类改革，深化人事制度改革和收入分配制度改革，加快推进社会保险制度改革，全面启动集体林权制度配套改革，继续抓好国有企业和集体企业改制，以改革促发展、促稳定、促和谐。大力支持民营经济发展，认真落实国家支持小微企业发展的各项政策措施，凡是法律未禁领域都允许民营企业进来，鼓励民营企业进入公路、城建、旅游、金融、公用事业等领域，推动形成我市民营经济新的发展高潮。全面扩大对外开放，进一步加大招商引资力度。要主动跟进，精细服务，提高履约率和落地率，重点推动中博会、农博会已经签约项目的投资到位和开工建设，以项目落地的良好业绩带动招商引资取得更大成果。

九、创优环境

发展靠环境，环境促发展。要努力营造有利于发展的法制环境、市场环境和社会环境。

1、推进民主法制建设。认真执行人大及其常委会的决议决定，自觉接受其法律和工作监督，支持人民政协履行政治协商、民主监督、参政议政职能，认真办理、落实人大代表的议案、建议和政协委员的提案，主动听取和采纳民主党派、工商联、无党派人士的意见建议，支持人民法院、检察院的工作。扩大基层民主，加强基层依法治理。深入推进“六五”普法教育，形成浓厚法制环境。

2、加强精神文明建设。进一步加强社会公德、职业道德、家庭美德和个人品德建设，组织开展“当代吕梁英雄”、“吕梁道德模范”的评选和宣传活动，积极倡导知荣辱、讲正气、作贡献、促和谐的良好风尚。支持工会、共青团、妇联等人民团体发挥更大作用，加强军政军民团结和“双拥”工作，重视外事、侨务、对台工作，办好民族宗教事务，充分调动各个方面的积极因素，形成推动吕梁转型跨越发展的强大合力。

3、创造良好社会环境。要进一步解放思想，更新观念，在全社会形成开放包容、合作共赢、支持创业、鼓励创新的良好氛围。要向一切投资创业者提供最大限度的支持和便利，对吃拿卡要的公务人员、对敲诈勒索的黑恶势力要给予从严、从重、从快打击。坚决制止公路“三乱”。要积极为企业生产经营提供全方位、现代化的服务，已协调好今年要引进太原海关在吕梁设立办事机构，使我市的进出口企业在本地就可以方便快捷地报关，把对外开放提升到一个新水平。同时，高度重视环境保护工作，在全市开展以“净空、净水、减排”为重点的绿色生态工程攻坚行动，特别要抓好污染减排工作，坚决淘汰落后产能，强化重点行业、重点项目减排工程管理，确保减排目标顺利完成。实现市区、各个县城生活污水处理和垃圾无害化处理全覆盖，在吕梁市区开展细颗粒物（PM2.5）监测。吕梁市区要创建省级环境保护模范城。

各位代表，软环境是经济发展的硬条件。我们要无比珍惜来之不易的大好局面，精心培育更加宽松优美的发展环境。只有这样，才能吸引来全国乃至世界的人才和投资，才能更加焕发出吕梁370万人民的豪情和智慧，才能使吕梁大地成为创业的乐园和发展的高地！

十、政府建设

围绕建设服务政府、效能政府、法治政府和廉洁政府的总目标，坚持以人为本、执政为民的理念，全面提高各级政府履职水平，为转型跨越发展提供有力保障。

1、强化政府服务职能。在加强和改善经济调节和市场差距的同时，更加注重社会管理和公共服务，为各类市场主体和人民群众提供更加公平、更为全面的政府服务。加大政务公开力度，自觉接受社会监督。办好政务大厅和公共资源交易中心，加快市县行政服务中心建设。

2、努力提高行政效能。进一步清理和规范行政审批事项，认真落实首问负责制、限时办结制、服务承诺制、责任追究制，探索审管分离，规范审批流程，推进并联审批，缩短审批时限。强化绩效管理，健全绩效考核指标体系和激励约束评价机制，加大行政监察力度，执行问责、问廉、问效制度，有责必问，有错必究，促进政府各部门依法行政、高效运转。

3、坚持做到依法行政。健全完善重大问题决策程序和规则，促进依法决策、科学决策、民主决策。强化审计监督和行政执法监督，认真落实执法责任制、执法责任追究制，坚决纠正执法不公、侵权害民等问题。

4、切实加强廉政建设。深入开展廉政建设和反腐败斗争，严厉查处各类违法违纪案件。坚持艰苦奋斗、勤俭节约，坚决反对铺张浪费，切实把有限的资金和资源用在改善民生的关键环节上。

5、建设一个执行力强的政府。凡是决策决定了的事，要雷厉风行、一抓到底；凡是承诺了老百姓的事，要按期完成，不折不扣。真正做到抓铁有痕，掷地有声，敢负责、敢担当、重落实，想干事，能干成事，成为人民群众信得过的、满意的政府。

各位代表，吕梁的土地是英雄的土地，吕梁的人民是英雄的人民，实践将会证明，我们吕梁人民有智慧、有能力，会把吕梁的事情办得更好，会把我们吕梁建设的更加富裕、更加文明。

各位代表，2011年的辛勤耕耘收获了“十二五”的良好开局，2012年的光荣使命承载着党和人民的殷切期望。让我们在中共吕梁市委的坚强领导下，万众一心，埋头苦干，锐意进取，扎实工作，不断开创转型跨越发展新局面，努力夺取各项工作新胜利，以优异的成绩迎接党的十八大胜利召开！
